# Supplementary material for: Multi-responsive chitosan-based hydrogels for controlled release of vincristine
Source: Commun Chem. 2023 Feb 10;6:28. doi: 10.1038/s42004-023-00829-1 (PMC9918727; doi:10.1038/s42004-023-00829-1)
Supplement: Supplementary file 2 — Supplementary Information [file 42004_2023_829_MOESM2_ESM.docx]

**Multi-Responsive Chitosan-Based Hydrogels for Controlled Release of Vincristine**

**Supplementary materials**

**Tables and ^1^HNMR spectra of prepared hydrogels**

**Table S1.** Particle size, poly dispersity index and zeta potential of drug-free and drug-loaded hydrogels

| Formulation type |  | Parameters, mean±SD (N=3) | | |
| --- | --- | --- | --- | --- |
|  |  | Size  (nm) | Zeta Potential  (mV) | Polydispersity Index (PDI) |
| CS-g-gly |  | 84.5±6.1 | 57.4±1.1 | 1.219±0.04 |
| CMCS-g-gly |  | 87.6±5.3 | 50.0±1.3 | 1.573±0.03 |
| VCR/CS-g-gly |  | 227.8±11.2 | 48.0±1.7 | 3.528±0.09 |
| VCR/CMCS-g-gly |  | 266.7±13.8 | 48.9±1.2 | 3.794±0.11 |

Abbreviations: PDI, polydispersity Index

**Table S2.** Entrapment Efficiency (EE%) of VCR/Cs-g-gly and VCR/CMCS-g-gly

| System | Carriers: Drug (mg/mg) | % STPP | Encapsulation Efficiency (%) |
| --- | --- | --- | --- |
| VCR/Cs-g-gly | 5:1 | 0.5 | 79.83 |
|  |  | 1 | 73.64 |
|  | 10:1 | 0.5 | 89.97 |
|  |  | 1 | 80.40 |
|  | 15:1 | 0.5 | 84.97 |
|  |  | 1 | 78.15 |
|  | 25:1 | 0.5 | 81.36 |
|  |  | 1 | 72.28 |
| VCR/CMCS-g-gly | 5:1 | 0.5 | 69.42 |
|  |  | 1 | 63.74 |
|  | 10:1 | 0.5 | 70.58 |
|  |  | 1 | 62.20 |
|  | 15:1 | 0.5 | 71.91 |
|  |  | 1 | 60.44 |
|  | 25:1 | 0.5 | 69.23 |
|  |  | 1 | 56.97 |

**Table S3.** The kinetic release models and the parameters obtained for the carriers.

| Release Model | Equation | (a)  pH=5 | (b)  pH=7.4 | (c)  pH=5 | (d)  pH=7.4 |
| --- | --- | --- | --- | --- | --- |
| Zero-Order | Ct=C0+K0t | R^2^= 0.8349 | R² = 0.7087 | R^2^=0.7937 | R^2^=0.6338 |
| First-Order | LogC=LogC0+Kt/2.303 | R^2^= 0.9315 | R² = 0.7422 | R^2^=0.798 | R^2^=0.628 |
| Higuchi | Q=KH$\surd t$ | R² = 0.5527 | R² = 0.4421 | R^2^=0.4953 | R^2^=0.3217 |
| Korsmeyer-Peppas | Mt/M =Ktn | R² = 0.9871 | R² = 0.5105 | R^2^=0.7676 | R^2^=0.7354 |
|  |  | n= 0.4291 | n=0.4413 | n=0.5475 | n=0.639 |

a) VCR/CS-g-gly at pH=5

b) VCR/CS-g-gly at pH=7.4

c) VCR/CMCS-g-gly at pH=5

d) VCR/CMCS-g-gly at pH=7.4

**Table S4.** Previous studies of vincristine drug delivery with different carriers

| No | Drug carrier | EE% | Release kinetic | Max amount release | Cell-tested | concentration | Incubation time | Cell viability | *In vivo* study | Ref |
| --- | --- | --- | --- | --- | --- | --- | --- | --- | --- | --- |
| 1 | dextran sulfate complex solid lipid nanoparticles | 93% | 144h | 92% | MDA-MB-231 cells | 250 ng/mL | 72h | 93% | Yes | [7] |
| 2 | Collagen-Chitosan Complex Film | 79.0 ± 1.0)% | 28day | 41 ± 2.0)% | N/A | N/A | N/A | N/A | No | [18] |
| 3 | silk gels | N/A | 32day | (49.82% ± 1.82%) | Human neuroblastoma KELLY cells | N/A | N/A | N/A | yes | [61] |
| 4 | PLGA-b-PEG nanoparticles | 6.01 ± 0.23 (DL) | 24h | 79.13 % | HepG2 | 0.205µM | 24h | 47.37 % | No | [62] |
| 5 | Chitosan Capped Silver Nanoparticles | 47.73±1.40% | 72h | 20 | A549 cells, MDCK cells | 50 µg/mL | 24h | 92.68 ± 0.36, 91.02 ± 0.83 respectively | No | [63] |
| 6 | folic acid–chitosan conjugated | 81.25 | 8h | 11.11% | NCI-H460 cells | 5 (mg/mL) | 24h | 46.47% | No | [17, 64] |
| 7 | folic acid–conjugated PLGA–PEG nanoparticles | 46.7 ± 6.2 | 60h | 78% | MCF-7 | 100 µg/mL | 24 h | 89.65 ± 0.84% | No | [65] |
| 8 | silk gels | N/A | N/A | N/A | Human Ewing sarcoma cells A673 | 1 ng/mL | 48h | 41% | Yes | [66] |
| 9 | liposomes | 90 | N/A | N/A | N/A | N/A | N/A | N/A | No | [67] |
| 10 | liposomes | 100 | 24h | 100 | L1210, B16/BL6 | N/A | N/A | N/A | Yes | [68] |
| 11 | Sphingomyelin-cholesterol liposomes | 95 | 72h | 25 | P388 and solid A431 tumours | N/A | N/A | N/A | Yes | [69] |
| 12 | silk fibroin foams | N/A | 21 day | 96% | human neuroblastoma KELLY cells | N/A | N/A | N/A | Yes | [70] |
| 13 | sonosensitive liposomes | 93 | 130 s | 88% | MCF-7 cells | N/A | 72h | N/A | Yes | [71] |
| 14 | vincristine sulfate-conjugated gold nanoparticles using liposomes | 99.2±0.1 | 24h | 90 | HeLa cell | N/A | 24h | N/A | Yes | [15] |
| 15 | vincristine sulfate-loaded poly (butylcyanoacrylate) nanoparticles | 57.58 ± 0.77% | 24h | 100 | Raji cells | N/A | N/A | N/A | Yes | [72] |
| 16 | PEG-PLGA | 68.2% | 13d | 81 | N/A | N/A | N/A | N/A | No | [73] |
| 17 | VCR-loaded lipid-polymer hybrid nanoparticles | 90% | 3 days | N/A | raji cells , a20 cells and hUVec cells | 100 µg/mL | 72h | 20%, 28% and 56% respectively | Yes | [74] |
| 18 | PLGA Nanoparticles | 70.92 3.78% | N/A | N/A | L-02, SMMC7721 ,BEL7402 and BEL7402/5-FU human hepatocarcinoma cell lines | 2 mg/mL | 48h | N/A | Yes | [75] |
| 19 | solid lipid nanoparticles (SLNs) and nanostructured lipid carriers (NLCs) | 84.5 ± 4.6 and 85.4 ± 2.8 | 60h | 80% and 79% respectively | U87 MG cells | N/A | N/A | N/A | Yes | [76] |
| 20 | lipid-polymeric nanocarriers | 83.1 ± 3.3 | 7days | 80% | raji cells | 20 µg/mL | 72h | 31% | Yes | [77] |
| 21 | Modified Liposome |  |  |  | CEM cells, Ramos cells | 100 nM | 24h, 48h | 36% (24h), 20% (48h)  and 37% (24h), 34%(48h) respectively | Yes | [78] |
| 22 | dextran microspheres amalgamated with thermosensitive gel | 60.4 ± 4.5% | 72h | 95% | THP-1 cells | 10 nM | 24,48,72h | 60%, 53% and 24% respectively | Yes | [79] |
| 23 | nanoliposome | 92 | 24h | 100% | The human cell lines Daoy (medulloblastoma) and SW480 (colon cancer) cells | N/A | 72h | N/A | Yes | [80] |
| 24 | pH-sensitive PLGA-PEG-folate and cell penetrating peptide R7-conjugated PLGA-PEG | 52.8 ± 3.5 and 48.9 ± 4.4 respectively | 60h | 89% | MCF7 cells,  MCF-7/Adr cell | N/A | 48h | N/A | Yes | [81] |
| 25 | microemulsions composed of PEG-lipid, oleic acid, vitamin E and cholesterol | N/A | 12h | N/A | N/A | N/A | N/A | N/A | Yes | [82] |
| 26 | F56 peptide conjugated nanoparticles | 21.4% | 30-day | 65.5% | HUVEC, cells CT-26 lung metastasis cells  HCT-15 cells | 100 nM | 24h | 24% | Yes | [83] |
| 27 | Pegylated Liposomal | 100.0% | 30 min | N/A | RM-1 cells | N/A | N/A | N/A | Yes | [84] |
| 28 | vincristine–oleic acid ion-pair complex loaded submicron emulsion | 78.64% ± 3.44% | 48h | 57% | MCF-7 | 0.1 µg/mL | 24h | 67% | No | [85] |
| 29 | Disulfide Cross-linked Micelles | 100% | 24h | 52% | Raji | 125 nM | 72h | 44% | Yes | [86] |
| 30 | liposome formulation | 78.3% | 5h | 50% | JIMT-1 cells | N/A | 24h | N/A | Yes | [87] |
| 31 | Liposomes | 97.92± 0.85 | 24h | 15% | MDA-MB-231 cells | N/A | 24h | N/A | Yes | [88] |
| 32 | 2-Hydroxyethyl Methacrylate Hydrogels | N/A | 24h | 100% | N/A | N/A | N/A | N/A | NO | [89] |
| 33 | silk fibroin hydrogel | N/A | 40 days | 10% | N/A | N/A | N/A | N/A | No | [90] |
| 34 | transferrin mimetic small peptides conjugated to vincristine liposomes | 91.6 ± 2.1 | 48h | 47% | GL261 cell line | 1 µg/mL | 48h | 43% | Yes | [91] |
| 35 | injectable-solid β-hairpin peptide hydrogel | N/A | 28 day | N/A | N/A | N/A | N/A | N/A | N/A | [92] |
| 36 | silk film | N/A | 20 day | 100 ± 6 | N/A | N/A | N/A | N/A | N/A | [93] |
| 37 | Novel synthesized chitosan-based hydrogel (VCR/Cs-g-gly) | 89.97% | 5 days | 82.27 | MCF-7, MCF-10 | MCF7 (50 ng/mL)  MCF-10 (50 mg/mL) | 24h, 48h | 35.30 (MCF-7-48h)  62.50 (MCF-10-48h) | No | This study |

**^1^HNMR spectra of prepared hydrogels**

^1^HNMR raw data of CS-g-gly

^1^HNMR raw data of CMCS-g-gly
