# Supplementary material for: Multi-responsive chitosan-based hydrogels for controlled release of vincristine
Source: Commun Chem. 2023 Feb 10;6:28. doi: 10.1038/s42004-023-00829-1 (PMC9918727; doi:10.1038/s42004-023-00829-1)
Supplement: Supplementary file 3 — Reporting Summary [file 42004_2023_829_MOESM3_ESM.pdf]

nature portfolio

Corresponding author(s): Sharzad Javanshir and Mohammad Reza Naimi Jamal

Last updated by author(s): Jan 22, 2023

# Reporting Summary

Nature Portfolio wishes to improve the reproducibility of the work that we publish. This form provides structure for consistency and transparency in reporting. For further information on Nature Portfolio policies, see our Editorial Policies and the Editorial Policy Checklist.

## Statistics

For all statistical analyses, confirm that the following items are present in the figure legend, table legend, main text, or Methods section.

| n/a | Confirmed | |
|---|---|---|
| ☒ | ☐ | The exact sample size ($n$) for each experimental group/condition, given as a discrete number and unit of measurement |
| ☒ | ☐ | A statement on whether measurements were taken from distinct samples or whether the same sample was measured repeatedly |
| ☐ | ☒ | The statistical test(s) used AND whether they are one- or two-sided<br>*Only common tests should be described solely by name; describe more complex techniques in the Methods section.* |
| ☒ | ☐ | A description of all covariates tested |
| ☒ | ☐ | A description of any assumptions or corrections, such as tests of normality and adjustment for multiple comparisons |
| ☒ | ☐ | A full description of the statistical parameters including central tendency (e.g. means) or other basic estimates (e.g. regression coefficient) AND variation (e.g. standard deviation) or associated estimates of uncertainty (e.g. confidence intervals) |
| ☐ | ☒ | For null hypothesis testing, the test statistic (e.g. $F$, $t$, $r$) with confidence intervals, effect sizes, degrees of freedom and $P$ value noted<br>*Give P values as exact values whenever suitable.* |
| ☒ | ☐ | For Bayesian analysis, information on the choice of priors and Markov chain Monte Carlo settings |
| ☒ | ☐ | For hierarchical and complex designs, identification of the appropriate level for tests and full reporting of outcomes |
| ☒ | ☐ | Estimates of effect sizes (e.g. Cohen's $d$, Pearson's $r$), indicating how they were calculated |

*Our web collection on statistics for biologists contains articles on many of the points above.*

## Software and code

Policy information about availability of computer code

| | |
|---|---|
| Data collection | Our manuscript has not included software and codes. |
| Data analysis | Our manuscript has not included analysis. |

For manuscripts utilizing custom algorithms or software that are central to the research but not yet described in published literature, software must be made available to editors and reviewers. We strongly encourage code deposition in a community repository (e.g. GitHub). See the Nature Portfolio guidelines for submitting code & software for further information.

## Data

Policy information about availability of data

All manuscripts must include a data availability statement. This statement should provide the following information, where applicable:

- Accession codes, unique identifiers, or web links for publicly available datasets
- A description of any restrictions on data availability
- For clinical datasets or third party data, please ensure that the statement adheres to our policy

Data are available upon request.

## Human research participants

Policy information about studies involving human research participants and Sex and Gender in Research.

| | |
|---|---|
| Reporting on sex and gender | Our manuscript has not included clinical trial. |
| Population characteristics | Our manuscript has not included clinical trial. |
| Recruitment | Our manuscript has not included clinical trial. |
| Ethics oversight | Our manuscript has not included clinical trial. |

Note that full information on the approval of the study protocol must also be provided in the manuscript.

# Field-specific reporting

Please select the one below that is the best fit for your research. If you are not sure, read the appropriate sections before making your selection.

☒ Life sciences ☐ Behavioural & social sciences ☐ Ecological, evolutionary & environmental sciences

For a reference copy of the document with all sections, see nature.com/documents/nr-reporting-summary-flat.pdf

# Life sciences study design

All studies must disclose on these points even when the disclosure is negative.

| | |
|---|---|
| Sample size | Our manuscript has not included in vivo or clinical life sciences experiments. |
| Data exclusions | Our manuscript has not included in vivo or clinical life sciences experiments. |
| Replication | Our manuscript has not included in vivo or clinical life sciences experiments. |
| Randomization | Our manuscript has not included in vivo or clinical life sciences experiments. |
| Blinding | Our manuscript has not included in vivo or clinical life sciences experiments. |

# Reporting for specific materials, systems and methods

We require information from authors about some types of materials, experimental systems and methods used in many studies. Here, indicate whether each material, system or method listed is relevant to your study. If you are not sure if a list item applies to your research, read the appropriate section before selecting a response.

### Materials & experimental systems

| n/a | Involved in the study |
|---|---|
| ☒ | ☐ Antibodies |
| ☐ | ☒ Eukaryotic cell lines |
| ☒ | ☐ Palaeontology and archaeology |
| ☒ | ☐ Animals and other organisms |
| ☒ | ☐ Clinical data |
| ☒ | ☐ Dual use research of concern |

### Methods

| n/a | Involved in the study |
|---|---|
| ☒ | ☐ ChIP-seq |
| ☐ | ☒ Flow cytometry |
| ☒ | ☐ MRI-based neuroimaging |

## Eukaryotic cell lines

Policy information about cell lines and Sex and Gender in Research

| | |
|---|---|
| Cell line source(s) | MCF-7 and MCF-10 cell lines were provided from cell bank of Pasteur Institute of Iran (IPI) |
| Authentication | Cell lines were identified by Pasteur Institute of Iran (IPI) |
| Mycoplasma contamination | all cell lines tested negative for mycoplasma |

| | |
|---|---|
| Commonly misidentified lines (See ICLAC register) | None to declare |

# Flow Cytometry

## Plots

Confirm that:

☒ The axis labels state the marker and fluorochrome used (e.g. CD4-FITC).

☒ The axis scales are clearly visible. Include numbers along axes only for bottom left plot of group (a 'group' is an analysis of identical markers).

☒ All plots are contour plots with outliers or pseudocolor plots.

☒ A numerical value for number of cells or percentage (with statistics) is provided.

## Methodology

| | |
|---|---|
| Sample preparation | All data about flow cytometry experiments are included in manuscript by details. |
| Instrument | All data about flow cytometry experiments are included in manuscript by details. |
| Software | All data about flow cytometry experiments are included in manuscript by details. |
| Cell population abundance | All data about flow cytometry experiments are included in manuscript by details. |
| Gating strategy | All data about flow cytometry experiments are included in manuscript by details. |

☒ Tick this box to confirm that a figure exemplifying the gating strategy is provided in the Supplementary Information.
